# A theory-based model of cumulative activity

**DOI:** 10.1038/s41598-022-18982-3

**Published:** 2022-09-17

**Authors:** Kole Phillips, Kevin Stanley, Daniel Fuller

**Affiliations:** 1 Department of Computer Science, University of Saskatchewan, Saskatoon, Canada; 2 Department of Community Health and Epidemiology, University of Saskatchewan, Saskatoon, Canada

**Keywords:** Risk factors, Public health

## Abstract

Energy expenditure can be used to examine the health of individuals and the impact of environmental factors on physical activity. One of the more common ways to quantify energy expenditure is to process accelerometer data into some unit of measurement for this expenditure, such as Actigraph activity counts, and bin those measures into physical activity levels. However, accepted thresholds can vary between demographics, and some units of energy measurements do not currently have agreed upon thresholds. We present an approach which computes unique thresholds for each individual, using piecewise exponential functions to model the characteristics of their overall physical activity patterns corresponding to well established sedentary, light, moderate and vigorous activity levels from the literature. Models are fit using existing piecewise fitting techniques and software. Most participants’ activity intensity profile is exceptionally well modeled as piecewise exponential decay. Using this model, we find emergent groupings of participant behavior and categorize individuals into non-vigorous, consistent, moderately active, or extremely active activity intensity profiles. In the supplemental materials, we demonstrate that the parameters of the model correlate with demographics of age, household size, and level of education, inform behavior change under COVID lockdown, and are reasonably robust to signal frequency.

## Introduction

Human locomotion and energy expenditure are fundamental to many of the social, medical, and biological sciences^1^. Energy expenditure underlies diet^2^, sedentary behaviour, and physical activity guidelines^3^. The effects of sedentary behaviour and physical activity on multiple health outcomes are well documented^4–6^. Distinct from the discipline of GIScience, which is concerned with where we move^7^, energy expenditure studies examine how, when, and how much we move. Modeling how we expend energy can inform research examining general health impacts of energy expenditure^8,9^, cities and health^10^, or training programs for elite athletes^11^.

Obtaining data to inform, ground, and test these models has been limited by technology^12,13^. Traditional studies into energy expenditure have focused on expensive but valid methods including doubly labelled water^1^. The detailed measurements required for these methods are usually constrained to laboratory settings, which can only stylistically represent the overall energy expenditure of free living situations. Over the last decade, researchers have increasingly turned to accelerometry to scale measurement to the population level and increase external validity^14–16^.

Acceleration, by definition imparts a force, and therefore expends energy, making accelerometry a method for estimating energy expenditure. Accelerometers are the most common tools used to measure human movement in free-living conditions^14^. Unprocessed acceleration data must be preprocessed to isolate the signal of acceleration specific to human movement^17^. Optimal methods for this computation continue to be a subject of academic discourse^18^. Once the accelerometer data has been processed, it is necessary to convert units of energy, of which the (proprietary) Actigraph counts are the most common, but open source measures are also employed^19–21^.

Energy expenditure models derived from accelerometry data typically group the continuous energy expenditure data into ordinal classes established in the literature, typically sedentary (SED), light activity (LA), moderate activity (MA), or vigorous activity (VA). A cut-point approach, common in physical activity research^12^, applies thresholds to define categories of physical activity intensity by population group (e.g., children, adults, older adults). Examples include Freedson cut-points for Actigraph counts among adults^22^, Troiano cut-points for Actigraph counts among adults^23^, or other cutpoints for older adults^24^. While cut-point approaches are common and have been extensively used in research, the approach has been the subject of critique. In particular, cutpoint approaches vary by population group and at a the individual level, including gender^25^, aerobic fitness status^26^, and clinical status^27^. Applying a cut-point universally to a data set makes an often unwarranted assumption of population homogeneity to facilitate model interpretability.

Machine learning models are also commonly employed for energy expenditure prediction, relying on multiple features derived from the raw accelerometer signals^28^ and the application of different machine learning models^29^. These features are fed into machine learning models that are designed to either classify energy expenditure categories or predict energy expenditure as a continuous variable. A recent systematic review shows that k-means is the most commonly applied machine learning model in the literature^30^. In general, machine learning models are often better at predicting sedentary behaviour and physical activity intensities than cut-point based approaches^29,31^. Machine learning methods have disadvantages, requiring labeled data, retraining against new data sources, and requiring more computational time^30^.

In this paper, we address current shortcomings of both cut-point and machine learning based models by developing a new model that is physiologically grounded, continuous in energy expenditure, and sensitive to individual differences. We demonstrate empirically that the vast majority of human cumulative energy expenditure can be described by a set of exponential decays in probability corresponding to the four intensities of energy expenditure (SED, LA, MA, VA) described in existing literature, leading to a single parametric distribution describing individual activity. An individual model can be created by applying a piecewise fit to individual data, with initial guesses for the break points set from established values in the activity intensity in the literature. The proposed model works exceptionally well, providing R^2^ values in excess of 0.9 for at least 90% of participants drawn from two large accelerometry studies. The model fits for both counts per minute and MIMS units, with no observable loss of accuracy. The model provides a parametric, continuous representation of aggregate energy expenditure for most people. By examining the behavior of the model we were able to further derive four classes of individual behavior from the data, which we call activity intensity profiles: participants whose activity matched existing theory well, participants who did not exhibit vigorous activity, participants for whom there was not difference between low and moderate activity, and participants who had a notable peak in very vigorous activity. These emergent activity intensity profiles constitute an additional contribution of our work.

## Results

**Figure 1:**
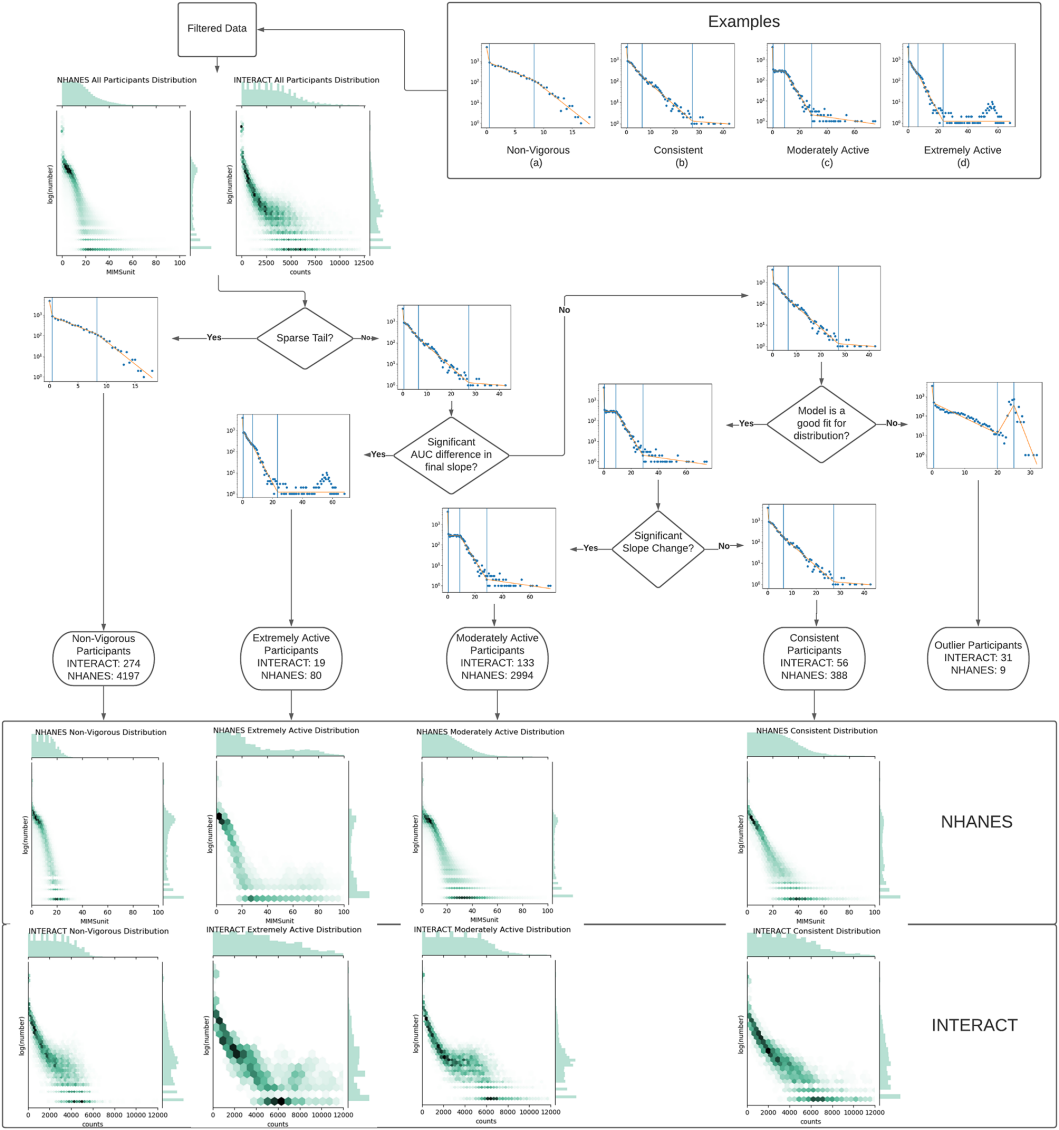
The process in which we classify participants after generating a model for them. Participants are assigned one of five activity intensity profiles based on the shape of their energy expenditure distribution. A participant is considered to be non-vigorous if they possess fewer than five data points in the vigorous activity range. If the participant’s vigorous activity level has a significant deviation between the area under the curves of the model’s slope and the actual data points, the participant is considered to be extremely active. If the participant has neither a Non-Vigorous nor an Extremely Active activity intensity profile and they have an R^2^ value of less than 0.9, they are classified as an outlier. The remaining participants are then sorted into Consistent if there is no significant change between the second and third slopes of the participant, or Moderately Active if there is a change.

Several filtering steps were applied to both datasets to ensure data quality. Participant and valid minute counts at each filtering step are provided in Table 1. Figure 1 provides a flowchart of the data analysis process with exemplar data.

**Table 1:** Number of participants and minutes remaining after each step in filtering.

	INTERACT		NHANES	
	Participants	Minutes	participants	minutes
Unfiltered (Initial Data)	548	4,084,674	7776	88,221,604
First Filter (Insufficient data)	513	3,972,035	7675	87,974,284
Second Filter (Activity count cap)	513	3,971,596	–	–
Third Filter (Remove tail outliers)	513	3,971,534	7675	87,966,989
Fourth Filter (Insufficient distribution points)	513	3,971,534	7668	87,890,587

The modeling approach proposed here posits that cumulative energy expenditure as measured by hip worn accelerometers can be well represented by a piecewise exponential decay, accounting for individual variability in cut-points and the rate of decay. Employing the piecewise linear fitting algorithm from^32^ on histograms of log transformed cumulative activity we can transform the empirical histogram into a parametric distribution. This model corresponds to a piecewise exponential distribution, where the components of the piecewise distribution are analogous to different activity intensities (sedentary, light, moderate, and vigorous activity), that are often operationalized using cut-points based on Actigraph counts in the literature^12,22–24^.

Under this model, once the cut-point has been defined, the probability of increased intensity (probability) of activity within an activity level decays exponentially with a given rate, dependent on the individual and activity level class. Piecewise fitting was applied to all 513 INTERACT and 7668 NHANES participants which remained after filtering. Because this model was fit using a piecewise linear algorithm on log transformed data, the quality of fit can be evaluated by the R^2^ value of the linear fit (shown in Fig. 3). Note that the probability axis is log-scaled, indicating that the distribution of R^2^ values are log-normal. Overall, the model fits exceptionally well for the majority of participants. For INTERACT data, 90% of the fits have an R^2^ of at least 0.9 and 99% have an R^2^ of at least 0.8, with a median R^2^ of approximately 0.95. For NHANES data 99% of fits have a R^2^ of at least 0.9 and 99.9% have an R^2^ of at least 0.8, with a median fit value of approximately 0.96. Note that the final bin contains very high R^2^ values, but none that are identically 1. The large number of R^2^ values in excess of 0.9 for both counts/min and MIMS units provides confidence that the proposed piecewise exponential model represents energy expenditure probability.

Four distinct regions were identified, corresponding to sedentary, light, moderate, and vigorous activity, were detected and fit. Results for the INTERACT data based on Actigraph counts show that the range of cut-points we detect using the piecewise model are between sedentary and light (mean = 153.24, range − 20 to 443.22, standard deviation = 51.54), between light and moderate (mean = 1930.08, range − 800 to 2500, standard deviation = 410.43), and between moderate and vigorous (mean = 5444.07, range − 1900 to 7000, standard deviation = 878.69). For the NHANES data we showed that cut-points are between sedentary and light (mean = 0.62, range − 0.5 to 1.5, standard deviation = 0.25), between light and moderate (mean = 8.99, range − 2 to 20, standard deviation = 3.41), and between moderate and vigorous (mean = 25.60, range − 5 to 50, standard deviation = 5.77).

Rarely, the low activity region had a small positive slope, indicating increasing likelihood of activity, as shown in the Fig. 1c. Occasionally the change in activity rate decay between the low and moderate intensity was small, as shown in Fig. 1b. Sometimes, the data captured an individual who was sufficiently inactive to have never registered any vigorous activity, having a behavior better represented by a three piece model Fig. 1a. Rarely participants exhibited peaks in vigorous activity, indicating some kind of regular high intensity physical activity like high intensity interval training, Fig. 1d.

Because the examples in Fig. 1 are log-linear plots, every increment in counts/min or MIMS is exponentially less likely to occur. As energy expended during a study is the integral of that exponential decay, the rate of decay has a profound impact on total energy expenditure. In all examples, more than 90% of minutes are classified below vigorous.

**Figure 2:**
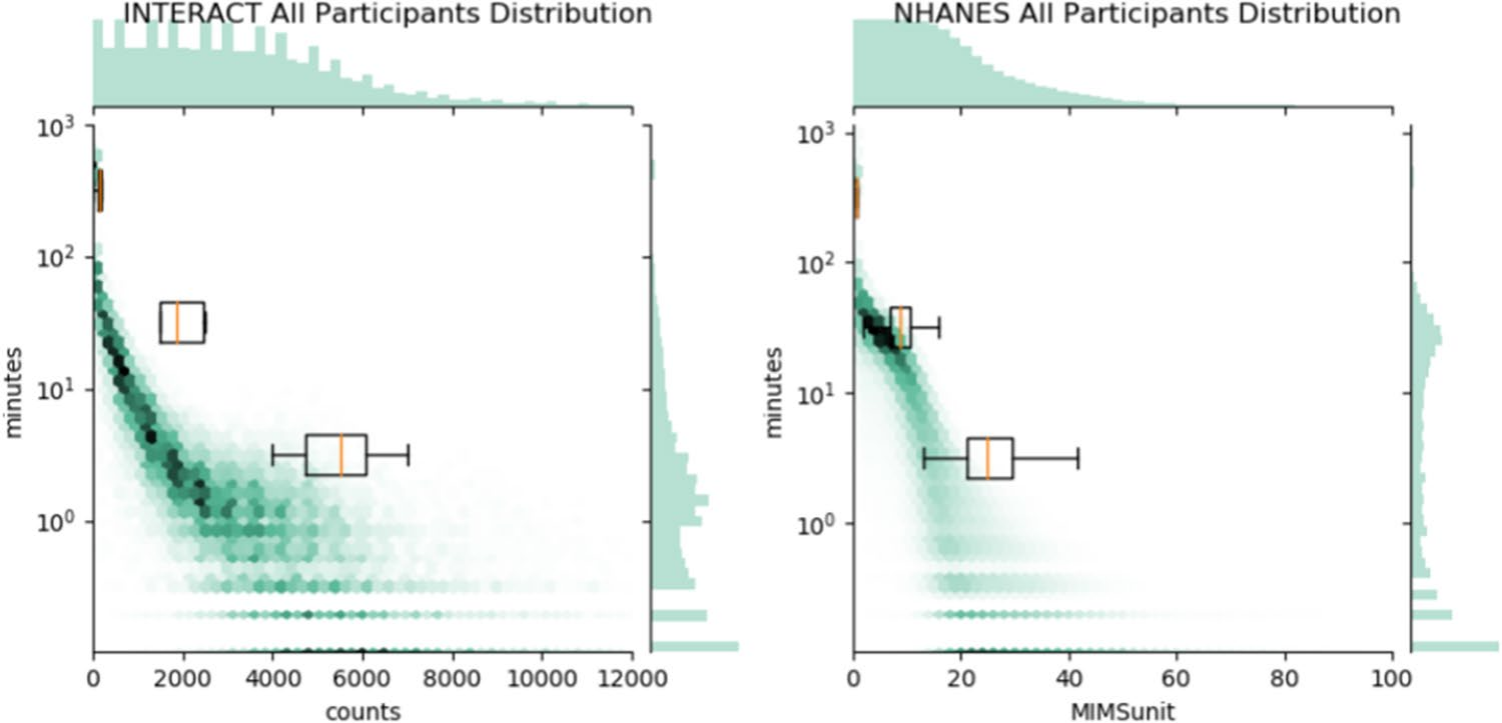
The distributions of all participants for the NHANES and INTERACT studies. The boxplots in each figure represent the distributions of each breakpoint, with the orange line representing the median, the box illustrating the upper and lower quartiles of each breakpoint, and the whiskers indicating the range of breakpoints.

**Figure 3:**
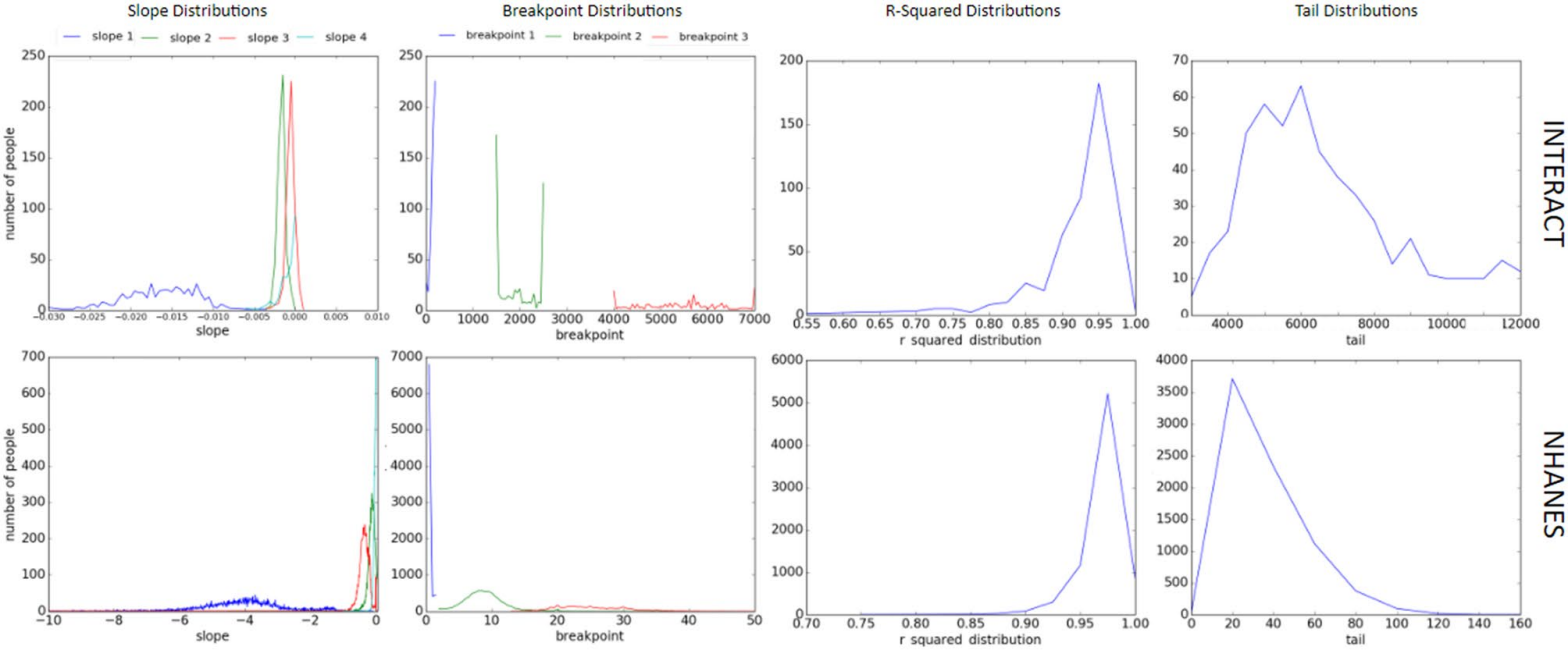
The distributions of the four slope metrics, the three breakpoint metrics, the R^2^ values, and the tail metrics for both INTERACT (top) and NHANES (bottom).

If we aggregate the per participant probabilities, one can create a heatmap of the likelihood of a participant having a likelihood of a specific activity rate. This heatmap visualizes the variability across the different activity rate regimes, showing where differences in activity levels for populations are likely to be evident. Figure 2 shows heatmap visualizations for the INTERACT and NHANES data. In both cases, sedentary behavior is largely consistent across participants. As activity rate increases the probability decreases, as expected, but the variance across participants increases. Figure 3 indicates, for the participants examined here, variation will be more apparent in the moderate and vigorous activity rate intensities. Both marginal count distributions indicate an exponential decay with energy expenditure. While at an individual level, there is variation in the rate of decay of energy expenditure with activity level, marginalized over the population, the result is a single exponential decay. Note that because counts/min and MIMS are not linearly related, we cannot directly compare the decay coefficients.

Because energy expenditure can be well represented by the model, we can examine the fit parameters of the model as descriptors of individual or population behavior. The model is fit to the log transformed data with the cut-points and exponential decay. Figure 3 shows the distribution of cut-points, exponential decay, R^2^ values, and maximum values for INTERACT and MIMS data.

The cut-points produced by our modeling approach are similar to Freedson cut-points for Actigraph counts among adults; SED (< 99 counts/min), LA (100–759 counts/min), MA (760–5724 counts/min), and VA (5725–max counts/min)^22^ and Troiano cut-points for Actigraph counts; SED (0–99 counts/min), LA (100–2019 counts/min), MA (2020–5998 counts/min), and VA (5999–max counts/min)^23^. Our modelled cut-points tended to be higher for the transition from sedentary to light activity and lower for the transition from moderate to vigorous activity. Critically, our cut-points are distributed across the population, accounting for individual differences. These differences could be due to inconsistency in lab based protocols used to develop previous cut-points and our free living samples. Differences could also be due to demographic differences between lab based studies and large population based samples used here. While individuals may exhibit different decay rates between light and moderate activity intensities, across the population, these rates are consistent, as was evident in the marginals in Fig. 3. The greatest variance is observed in the vigorous activity break point and maximum activity recorded, as individuals had a great deal of variability in their maximum achievable activity intensity. The variability in cut-points, but consistency in decay, indicate that the increasing variability in activity intensity seen in Fig. 3 may be due to a number of factors. Regardless of your absolute ability to carry out moderate and vigorous activity, there is limited time, motivation, or ability to do so for extended periods. We explore the possibilities of the days of the week and participant demographics being a factor in the supplemental materials.

## Discussion

The purpose of this paper was to develop and examine the utility of a generalized approach to understanding human activity intensity using accelerometer data. The approach uses concepts from exercise physiology including cut-points, and activity intensity categories (sedentary, light, moderate, vigorous activity). The piecewise log-linear model works exceptionally well, providing R^2^ values in excess of 0.9 for 415 INTERACT and 7615 NHANES participants (80.9% for INTERACT, 99.3% for NHANES). The supplemental material shows that the range of cut-points are plausible based on previous literature and that they follow expected patterns based on demographic characteristics. An interesting contribution of this model is the characterization of decay rates for activity intensity. Based on the exceptionally strong fit between model and data, we can conclude that activity intensity in humans is well modeled as piecewise exponential decay.

This work provides a definitive quantitative, parametric model of human mobility grounded in the physical activity and energy expenditure literature. The model accounts for individual differences in energy expenditure by allowing the breakpoints between sedentary, light, moderate and vigorous activity to be dictated by the data. When doing so, a piecewise exponential decay is noted for almost all participants, indicating a geometrically increasing inability to exert linearly more energy. This decreasing ability varies across individuals and between activity regimens. The existence of a more sedentary cohort who exhibit no vigorous activity is unlikely to be a surprise to health researchers.

We note from the model that there is a proportion of participants who have only a small change in the slope of the model between light to moderate activity. Given currently 24 h movement guidelines^33^, recommendations that more movement is better, and based on our findings, it may be the case, both from a human activity intensity perspective and from a health policy perspective, that having a strict distinction between the light and moderate physical activity categories is not always justified.

Some participants who engaged in extremely vigorous activity were not represented by the model’s final decay, and were assigned the “extremely active” activity intensity profile. These participants did not appear to engage in more moderate and vigorous activity and their exponential decays were similar to other participants for moderate to vigorous activity intensity. However, these participants then engaged in activities that previous cut-point literature would describe as extremely or very vigorous^24^. For these participants, the decay in intensity was similar to other participants up to the vigorous activity line segment, which contained a characteristic activity spike. For MIMS units, the median occurrence of this peak was at 59 units and for Actigraph counts the median peak occurred at 7900 units. Traditional analysis using the number of minutes of moderate to vigorous physical activity would classify these participants as active, but would miss an important feature of their activity patterns. Nearly all of their total moderate to vigorous activity is extremely vigorous. This group could be important to examine in relation to the potential health benefits and risks of primarily doing vigorous activity.

In creating this model we have followed Occam’s principle, and accepted the simplest explanation—that the observed exponential decay was in fact representing an exponential decay. However, it is possible that the curve is also a variation on a log transformed normal curve where only the decay portion is evident for most participants. While this would help explain the existence of peaks in the habitual exercisers, it complicates the model, and it seems simpler to posit that exponential decay represents normal life, and the peak in vigorous activity for a small number of participants represents a conscious behavior, with a different underlying process and distinct mathematical model.

We have analyzed the data across over 8000 participants from across North America, and found remarkably good agreement with the proposed model. Based on the quality of the fit, and the proportion of outliers, we expect that the model will generalize to most of the developed world. We expect that there would be exceptions, for example Maasai herders and American mail carriers are likely to have peaks similar to the habitual exercisers in the low to moderate activity range, given their lifestyles. By providing a model baseline and expected behavior, we have a standard for more distinct populations to be measured against. The methodology of fitting piecewise curves to populations would still hold. However, care must be taken when tuning the meta parameters—the initial guesses and ranges for the cut-points between activity levels. Undue analyst intervention could bias the fit towards or away from a particular outcome. We expect that the slopes and distributions will be population dependent, and the slope and breakpoint values in Fig. 3 would not likely hold across the developed world. For example, cities like Paris or Tokyo, which have substantial and highly subscribed public transit systems, might see a slower decay in LA compared to residents of Atlanta, which has an entrenched car culture^34^.

While this work has provided a substantial and new model of human energy expenditure and behavior, it is not without shortcomings or opportunities for future work. The most important test for a new model is utilizing new data. Large studies like UK Biobank, which include accelerometer data but uses the Euclidean Norm Minus One method for raw accelerometer data processing should be reanalyzed using this model to ensure generalizability^35^. Targeted studies at populations likely to be outliers (like the herders and mail carriers mentioned earlier) should be undertaken to circumscribe the applicability of the model, and extend the model to these new populations. Both datasets were restricted to hip worn accelerometers. Future work should investigate the extent to which our findings hold to accelerometers worn in other locations such as the wrist. The pwlf algorithm should be extended to be less susceptible to outliers, and more likely to identify the activity regions with fewer meta parameters. More controlled validation studies should be undertaken with gold standard energy expenditure measures such as direct or indirect calorimetry to tease out the interplay between physiological and psychological drivers which underlie this model. We have attempted to illustrate potential applications of this technique to common problems in the literature including comparing stratification by demographic variables in the supplemental material. However, these preliminary studies are meant to be illustrative of technique rather than conclusive in findings. Further studies exploring the utility of the technique are warranted. Finally, there exists a substantial opportunity to employ this model in energy expenditure studies, particularly in health, where the model can be used to compare individuals or populations with respect to an intervention based on changes to the model parameters including the slopes and breakpoints.

## Conclusion

Energy expenditure is a fundamental component of many health, social and biological studies. Previous studies have been limited by the temporal resolution of the data and the methodologies developed to analyze those data. We have proposed a new method of characterizing human energy expenditure measured by accelerometry, which employs a piecewise log-linear fit to account for individual differences. Based on our analysis most participants analyzed can be characterized by piecewise exponential decays in energy expenditure, with the decay constants and breakpoints between energy expenditure regimes differentiated between individuals. This model provides a more complete and precise method of accounting for individual and group energy expenditure than previously reported in the literature.

## Methods

### Data

The INTerventions, Research, and Action in Cities TEAM (INTERACT) study is a built environment natural experiment study in four Canadian cities; Victoria, Vancouver, Saskatoon, and Montreal, and under ethics approval from Simon Fraser University (2017s0158, 2017s0531, and 2018s0127), the University of Saskatchewan (17-347), the Centre de Recherche du Centre hospitalier de l’Université de Montréal (CÉR CHUM 16.397), and Memorial University of Newfoundland (20180446)^36^. We include data from participants recruited who wore a SenseDoc (Mobysens) for 10 days during the first wave of data collection. The accelerometer was set to measure at 50 Hz continuously. The analysis sample included 548 participants, contributing a total of 4,084,674 min of data. On average participants contributed 10.62 days (range 2–20) days and 700.82 min (range 1–1440) per day of data. Ethical approval was given by the ethics board of Simon Fraser University, ensuring appropriate guidelines and regulations were followed while collecting data, and all involved participants provided informed consent. A more detailed characterization of the dataset can be found in^37^.

Publicly available data from the National Health and Nutritional Examination Survey (NHANES) physical activity monitor (PAM) were also analyzed^38,39^. Briefly, participants were asked to wear an ActiGraph GT3X+ (ActiGraph, Pensacola, FL) accelerometer, running continuously at 80 Hz, starting on the day of their exam in the NHANES Mobile Examination Center for 9 days. Our analysis uses the x-axis MIMS unit data at the minute level (PAXMTSM) from the publicly available data^39^. The total analysis sample included 7775 participants, contributing a total of 88,223,479 min of data. On average participants contributed 8.88 days (range 1–9) days and 1273.33 min (range 10–1440) per day of data. This data collection received informed consent from all participants and followed all necessary guidelines.

### Analyses

Computation occurred on the Cedar cluster of Compute Canada using Python 3.7.4, primarily using the libraries pwlf (2.0.4), scipy (1.3.1), pandas (0.25.1), and numpy (1.17.2). Figures shown in this paper were generated on a desktop PC with 15 GB RAM and an Intel Core i7-8700 processor, and were created through Python 3.7.4 using matplotlib (3.1.1) and seaborn (0.9.0). Performance characterization can be found in the Supplemental Materials.

**Figure 4:**
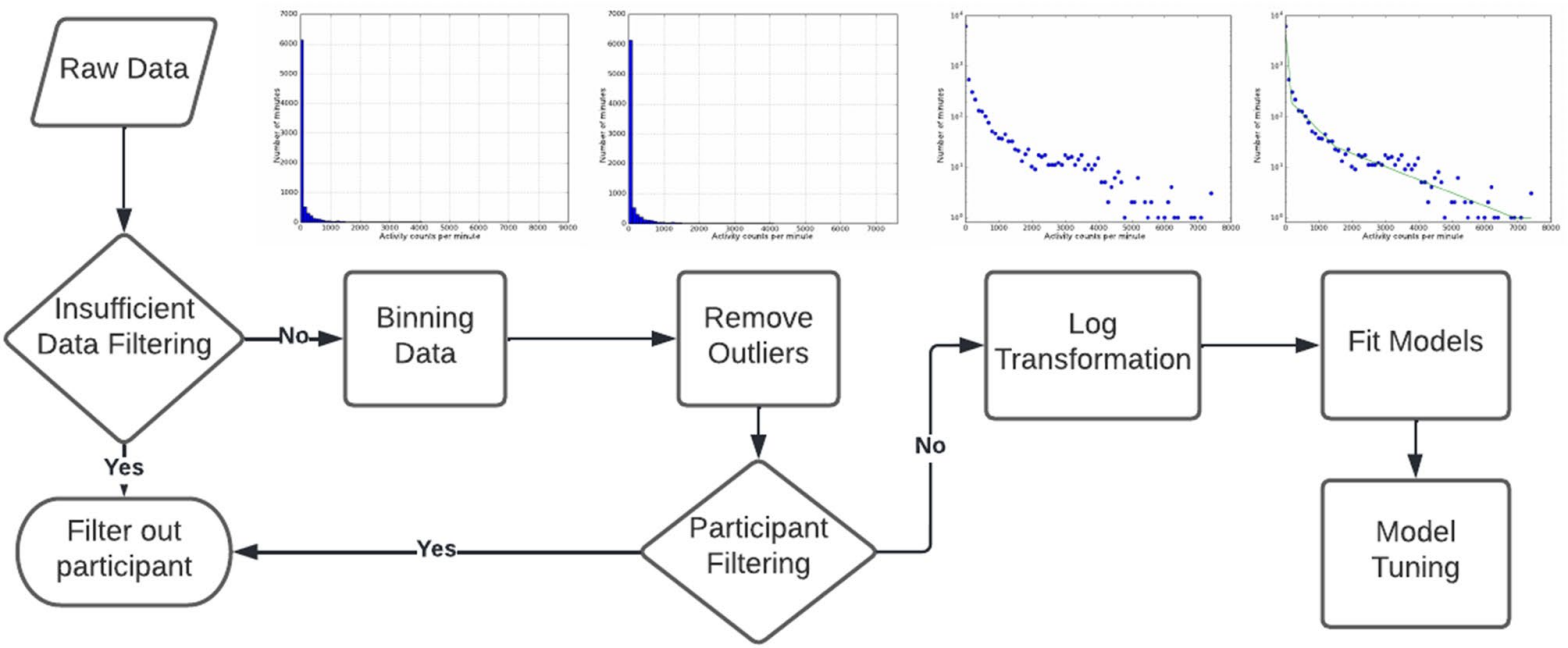
The process in which a participant is modelled. The plots above some stages in the process help visualize what a given participant’s data may look like at that particular stage.

The main steps to generate our model from a given set of telemetry data are shown in Fig. 4 and described below.

#### Data preparation

If a participant did not contribute data for at least 600 min per day of at least 3 different days, that participant was removed. We discuss how a lack of data can influence our model in the supplemental materials. To create an empirical activity intensity distribution, data from each participant was binned (bin width = 100 Actigraph counts/min or 0.5 MIMS). Participants with fewer than five non-zero bins were removed as outliers in this step. Actigraph counts from INTERACT data with more than 12,000 counts per minute were removed as physically implausible. After data was binned and initial outlier removal complete for insufficient data and extreme Actigraph counts, for each remaining participant, outlier data points were removed. These outlier points were detected by checking to see if there were any other data points within a number of units equal to 10 times the bin size we use when binning that unit’s data (thus, an outlier has no other data entries within 1000 activity counts or 50 MIMS units of itself). This process was repeated on the participant until a data point was not removed following this logic. The total number of participants and the total minutes of data filtered out at each of these steps can be found in Table 1. Initial data exploration indicated that a piecewise exponential distribution might be an appropriate model. To facilitate fitting a piecewise exponential model, the data points were transformed to their natural logarithms on the y-axis to allow linear fitting of transformed data.

Our core hypothesis was that cumulative human energy expenditure could be well described by a piecewise exponential distribution, with each component of the distribution corresponding with one of the four canonical exercise intensities (SED, LA, MA, VA). Fitting the exponential distribution based on existing cut-points is the core step in the analysis. The Python library pwlf is used to calculate piecewise linear fits^32^. The pwlf library is capable of determining the best breakpoints to fit a piecewise linear function given a set of data points. The pwlf library provides piecewise linear fits using one of two processes. The differential evolution algorithm (DE) is employed when no hypothesis about the approximate locations of breakpoints exist. When initial possible breakpoints between segments are known the Limited-memory Broyden-Fletcher-Goldfarb-Shanno (LBFGS) algorithm is used^40^. DE is more versatile but slower than LBFGS. LBFGS has the advantage of allowing a priori external information for the possible location of cut-points. We employed the LBFGS approach as substantial literature exists on the potential locations of Actigraph activity cut-points^12,22–24^ for INTERACT data, and an initial DE-driven data exploration to generate approximate breakpoints from a subset of the NHANES MIMS data, followed by LBFGS using those approximate cut-points for the whole NHANES sample.

Our model contains three breakpoints creating a model consisting of four segments, one for each activity intensity (sedentary, light, moderate, and vigorous activity). Drawing from existing Actigraph literature^12,22–24^, the first breakpoint is between 20 and 200 counts with a starting point of 100, the second is between 1500 and 2500 with a start point of 1951, and the third is between 4000 and 7000 with a start point of 5725. The MIMS unit had no established cut-points at the time of writing. The model was initially run on the NHANES dataset without any given breakpoint bounds or startpoints, using the DE algorithm. Breakpoint ranges and start points were derived from the median values returned for each breakpoint, and these ranges were optimized using LBFGS in later iterations. The R^2^ values of the model and log-transformed data were used to identify which iterations used the best breakpoint ranges and startpoints for the NHANES dataset. The resulting MIMS unit ranges we use are between 0.5 and 1.5 for the first breakpoint starting at 1.0, 2–20 for the second starting at 5, and 5–50 starting at 30 for the third breakpoint. It should be noted that these breakpoints were empirically derived, not based on validation studies using gold standard criterion measures as with Actigraph counts. However, these empirical values serve as a hypothesis for future validation experiments using MIMS units.

Based on our calculated metrics using the piecewise linear models, we divide participants into one of five different activity intensity profiles: non-vigorous, consistent, moderately active, extremely active, and outlier, as shown in Fig. 1. We follow a simple set of heuristics to categorize each participant. First, the model’s final segment is examined. If there are fewer than 5 points in that segment, we consider the participant to have a non-vigorous activity intensity profile. To represent non-vigorous participants, we use a 2-breakpoint 3-segment model because there was insufficient vigorous activity to model. The area under the curve (AUC) for both the original point distribution and our model’s representation of the distribution are calculated for all remaining participants’ vigorous activity. Extremely active participants have a characteristic lognormal vigorous activity level distribution. If the AUC of the vigorous model segment is 70 percent or less than the AUC of the corresponding empirical data points, the participant has an extremely active activity intensity profile. To lessen the impact of single outlier data points in the tail, the highest point in the tail is excluded from the distribution’s AUC. If a participant has not been classified as either non-vigorous or extremely active, we check the fit of the model to the distribution. If the model’s R^2^ value is less than 0.9, we mark that participant as an outlier. Finally, the remaining participants are assigned either moderately active or consistent activity intensity profiles. To differentiate these categories, we measure the angles of the slopes between the second and third line segments of the model. If the difference in angles of these lines exceeds 0.1 radians for MIMS unit readings or 0.001 radians for activity counts, we consider the participant to be moderately active, and consistent otherwise. The number of participants assigned to each classification can be found in Table 2.

**Table 2:** Number of participants with each activity intensity profile for both INTERACT and NHANES.

	Non-vigorous	Consistent	Moderately active	Extremely active	Outlier
NHANES	4197	381	2938	143	9
INTERACT	274	53	134	20	32

## Supplementary Information

Supplementary Information.

## Data Availability

We validate against two large accelerometry datasets, one embargoed (INTERACT in Actigraph counts)^36^ and one publicly available (NHANES in MIMS units, accessible from https://wwwn.cdc.gov/nchs/nhanes).

## Code availability

The code developed for this paper is located at the following Zenodo repository: https://zenodo.org/record/6336088#.YiaTNhvF05k.
