# Supplementary material for: A theory-based model of cumulative activity
Source: Sci Rep. 2022 Sep 17;12:15635. doi: 10.1038/s41598-022-18982-3 (PMC9482623; doi:10.1038/s41598-022-18982-3)
Supplement: Supplementary file 1 — Supplementary Information. [file 41598_2022_18982_MOESM1_ESM.pdf]

1 Supplementary Information

The true test of a model is its utility. In the supplementary information to this paper we provide a series of short case studies demonstrating how the model can be used. These case studies include; a) changes in behaviour during COVID-19, b) model resilience to lack of data, c) associations between participant demographics and activity intensities, d) comparison of weekday versus weekend behaviour, and e) computational runtime. These case studies provide evidence of this new models utility in producing results that are similar to what is known about weekly and demographic variations in activity intensity, and also provide evidence that the model can be implemented with relatively ease.

1.1 Appendix A: Changes in Behaviour During COVID-19

The COVID-19 pandemic resulted in a dramatic shift in behaviour for many individuals as lockdowns and other measures went into effect around the world. Studies using questionnaires and self-reported data have been conducted in several countries including Spain[1], Italy[2], and the United Kingdom[3], and all have shown physical activity levels have lowered dramatically since pre-pandemic times. A systematic review of studies across multiple populations, using self-report and accelerometer data showed that in 64 out of 66 studies included in the review physical activity declined during COVID-19 lockdowns[4]. Step count information taken from the fitness app Argus showed that the mean number of daily steps taken dropped by 27.3% within 30 days of the World Health Organization announcement of the COVID-19 pandemic[5].

The first wave of INTERACT data and was collected between May 31, 2017 and February 21, 2019 in four Canadian cities, Victoria, Vancouver, Saskatoon, and Montreal. The second wave of this study took place in 2019 and 2020, during the COVID-19 pandemic in Vancouver, Saskatoon, and Montreal. To see how this event impacted our metrics, we looked only at participants who took part in and provided sufficient data to both the first and second waves of data collection. Collecting data during the pandemic was extremely challenging with participants not wanting to return to the study, resulting in a significantly lower number of participants in the second wave. To compensate for the lower number of participants, we lowered our filtering thresholds from 600 minutes per day for three days to 200 minutes per day for three days for this analysis. There were a total of 50 participants who met these conditions.

Figures 1a and 1b show the distribution of all minutes among all common participants for INTERACT waves 1 and 2. Comparing these figures indicates that the peak physical activity of our returning participants is slightly lower than in the first wave of data collection. In wave 2, the distribution in the moderate and vigorous physical activity levels appear to be much flatter and closer to 0 when compared to wave 1. Table 1 compares how the 50 returning participants profiles change between wave 1 and wave 2, and it indicates that several participants who were somewhat active before the pandemic have become less so.

Table 1. INTERACT participant activity intensity profiles over two waves

	Non-vigorous	Consistent	Moderately Active	Extremely Active	Outlier
Wave 1	22	10	13	2	3
Wave 2	27	2	8	2	11

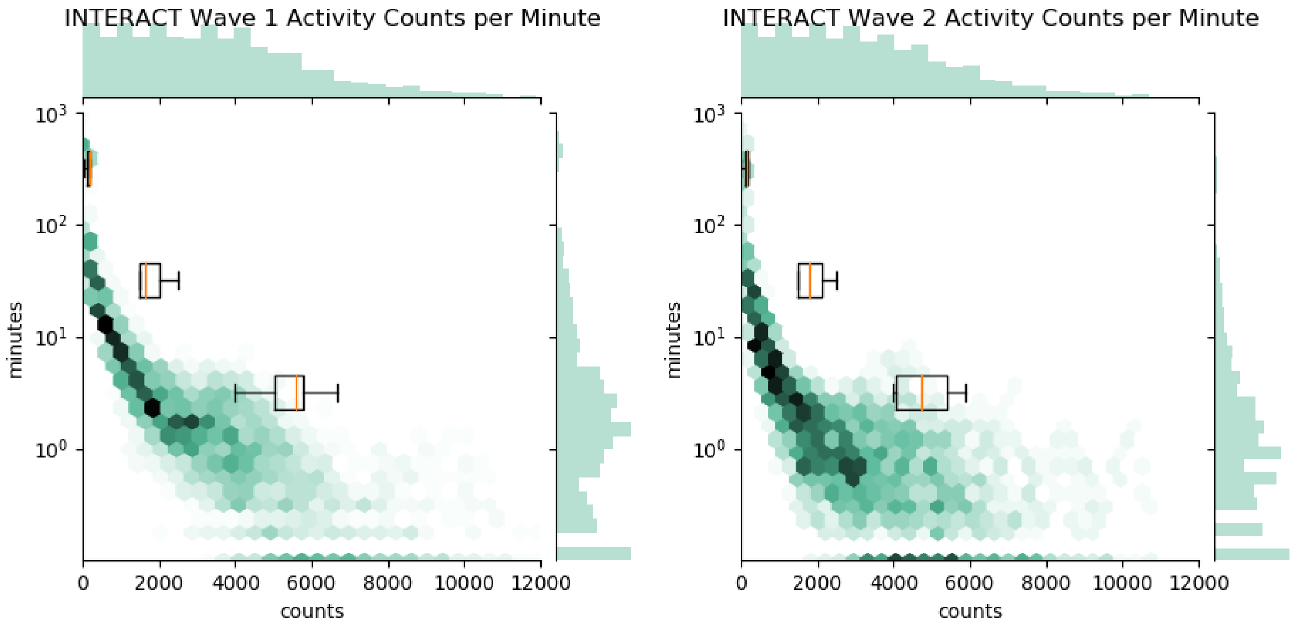

Figure 1. A comparison of the distributions of activity counts between INTERACT's first (a) and second (b) study waves, only taking into account the 50 participants who were present in both waves. The boxplots in each figure represent the distributions of each breakpoint, with the orange line representing the median, the box illustrating the upper and lower quartiles of each breakpoint, and the whiskers indicating the range of breakpoints.

1.2 Appendix B: Model Resilience to a Lack of Data

Data records are sometimes lost, through physical device failure, lack of participant compliance, or other factors. It is important to know how much of this data can be lost before a participant's data should be excluded from the study. Several studies have approached this problem by filling in missing records with existing records by assuming the records are lost at random and filling the records accordingly[6] or using complete records from similar individuals to model what sort of records would be produced at the times the records are missing[7]. Studies have also been performed to identify the minimum number of records to accurately represent physical activity levels using step counts[8] and accelerometer data[9]. Parametric models explicitly allow interpolation of missing data, as the derived function has continuous support. Our model could be used to replace missing data at the aggregate level. To characterize the degree to which the model could interpolate the data we performed a downsampling sensitivity analysis.

To observe the effects that a sparse dataset would have on our model, we created new datasets from the original physical activity datasets. A cycle of 1 minute kept, X minutes skipped was used to construct the X-minute level dataset, where the 0-minute level dataset is the original dataset and the values of X examined were 1, 2, 4, 8, 16, 32, and 64. At the 1-minute level only half of the original dataset is retained, at the 2-minute level only one third of the original dataset is retained, and so on. To avoid all participants being removed from the dataset in the filtering stages at higher values of X, and to allow the resulting metrics to be comparable to those of the original datasets, each minute that is skipped in an X-minute level dataset is instead overwritten by the previous minute that was kept. These datasets were constructed for both the INTERACT and NHANES datasets.

In Tables 2 and 3, we see the number of participants with each activity intensity profile in each of the X-minute level datasets, with Table 2 representing the NHANES dataset and Table 3 representing the INTERACT dataset, along with their X-minute level derivatives. Note that the total number of participants may vary between tables as some differences in the datasets may still cause some participants to be filtered out when they were not filtered out in the original dataset.

As seen in these tables, as more data is removed from the database, more the activity intensity profiles are non-vigorous, likely because the data points in the tail are usually the lowest frequency records for a given participant. As more and more data are removed from the database, it is increasingly likely that the majority of each participant's vigorous activity records are also lost, to the point that the participant has too few data points in the tail, and their activity intensity profile becomes non-vigorous. As the number of minutes skipped increases, the number of participants whose profiles are outliers also increases, although as shown in the tables below those participants' profiles eventually become non-vigorous once sufficient data is removed. In particular, we see that the extremely active activity intensity profiles rapidly deteriorate as early as the 1-minute (50%) level.

One notable trait which the NHANES dataset possesses but is not reflected in Table B-1 is that over 1000 participants with a non-vigorous profile in the original dataset have a moderately active profile in the datasets ranging from the 1-minute level to the 4-minute level. This is not reflected in the table because a much greater number of participants with a moderately active profile in the original dataset have a non-vigorous profile in those same datasets. This pattern is essentially nonexistent in the INTERACT data.

Figure 2 show the overall distribution for all participants in the both datasets for the original set, 1-minute level, 2-minute level, and 4-minute level datasets, as well as a boxplot marking the breakpoints for each participant's resulting model from that dataset, with the INTERACT plots appearing on the left and the NHANES plots appearing on the right. In both datasets, we see the tail decline and the points within the third slope of the model draw closer to 0 as more and more data is removed, which also demonstrates the rapid decline of the extremely active participants and the steady migration of participants to non-vigorous profiles. We see that as the dataset has a lower measurement frequency, the model underestimates the time spent performing vigorous activity.

Table 2. NHANES participant activity intensity profiles as data is skipped for every minute of data lost

Minutes Skipped	Non-Vigorous	Consistent	Moderately Active	Extremely Active	Outlier
0	4197	388	2994	80	9
1	4725	341	2579	11	11
2	5010	303	2344	4	4
4	5451	235	1956	2	17
8	5990	170	1431	1	65
16	6579	84	696	0	291
32	7120	11	93	0	417
64	7395	0	6	0	210

Table 3. INTERACT participant activity intensity profiles as data is skipped for every minute of data lost

Minutes Skipped	Non-vigorous	Consistent	Moderately Active	Extremely Active	Outlier
0	274	55	134	19	31
1	306	45	96	7	59
2	328	43	60	1	81
4	360	17	49	0	87
8	403	7	33	0	70
16	439	0	21	0	53
32	481	1	14	0	17
64	502	1	2	0	8

1.3 Appendix C: Participant Demographic and Activity Intensity Associations

How different demographic groups differ in terms of physical activity levels has been the topic of many studies. Marital status has been found to be an indicator of lowered activity levels, particularly among women[10, 11]. Studies have found that women in general have lower levels of physical activity when compared to men[11, 12, 13], that individuals tend to have lower physical activity levels as they get older[12, 13], and that education levels appear to correlate with in physical activity[11, 13].

During the NHANES study, a number of surveys were taken by the participants, including a demographics survey which included the participant's age in years, the participant's ethnicity, what education level the participant has attained, the number of individuals in the participant's household, the participant's gender, and the annual income of the participant's family. Not all participants in the dataset have a response recorded for every question.

To determine whether or not our metrics reflect the differences in participant demographics, we used an Analysis of Variance (ANOVA) to test the association between each of these demographics and our metrics. Of the metrics we produce, the activity intensity profile and maximum value appear to have the strongest correlation with the listed demographics. Of these demographics, age, education, and household size are associated with the breakpoint metrics across nearly all of the cases when examining the corresponding p-values. Table 4 shows the ANOVA coefficients for each decade of the age survey question. Table 5 shows the same corresponding values for the education levels of adults, while Table 6 show those values for household sizes.

Looking at Table 4, the most obvious correlation between age and our metrics is with the distribution's maximum value, which steadily decreases with each decade of the participant's life, meaning that the older a person is, the lower their maximum physical activity output. The model's second and third breakpoints follow this trend, while the first breakpoint has the opposite behaviour. Meaning that compared to those 0-19 years old, sedentary activity increases over time for each 10 years of age category, while vigorous activity decreases over time for each 10 years of age group, compared to the 0-19 group. Light and moderate activity increase until age 49, then decrease after age 49, when compared to the 0-19 age group. The first slope, which represents the rate of decay of sedentary activity, appears to increase with age, while the other three slopes do not appear to have a discernible trend when comparing age groups.

Referencing Table 5, we see the maximum activity tends to have a positive correlation with education level. The first slope and the second breakpoint also increase with higher education, while the second slope decreases. The remaining metrics do not appear to have a strictly positive or negative correlation with the individual's level of education.

When we compare the individual's household size to our metrics in Table 6, the maximum increases with the number of household members, while the first slope decreases. The third breakpoint and the second slope increase with household members, but this increase stops around 4 to 5 members, after which the values become relatively constant. The first slope is similar to the previous metrics, but decreases until 4 members. Compared to having one or two people in the household, the second and third breakpoints are greater for households with three or more people. Meaning that households with more people tend to have higher breakpoints for the separations between light and moderate, and moderate to vigorous activity. This could be due to households with more people tending to younger and likely including children. The remaining slope metrics once again did not appear to correlate with the number of people in the household.

Figure 4 is a line graph showing the ages of each of our activity intensity profiles. Each line represents what proportion of the age group has profiles that are non-vigorous, consistent, moderately active, and extremely active. The fraction of non-vigorous activity intensity profiles appears to be steady across all age groups except children, while the consistent and moderately active activity intensity profiles appear to be mostly dominated by children and young adults. The extremely active activity intensity profiles appear to have a large number of middle-aged members. Figure 3a shows how each activity intensity profile breaks down in terms of how many people live in their household, while 3b shows how each profile is broken down in terms of their education level.

Table 4. ANOVA test coefficients when comparing NHANES age responses to our metrics. Coefficients with corresponding p-values less than 0.05 are in bold.

Metric	0-19	20-29	30-39	40-49	50-59	60-69	70-79	80+
BP 1	**REF**	**0.06**	**0.07**	**0.05**	**0.08**	**0.11**	**0.16**	**0.18**
BP 2	**REF**	**1.22**	**0.66**	**0.63**	-0.15	**-0.36**	**-0.89**	**-1.50**
BP 3	**REF**	**-6.81**	**-7.21**	**-7.20**	**-7.88**	**-8.30**	**-10.1**	**-8.16**
Slope 1	**REF**	**1.04**	**1.19**	**1.44**	**1.60**	**1.90**	**2.15**	**2.36**
Slope 2	**REF**	**-0.10**	**-0.09**	**-0.09**	**-0.08**	**-0.10**	**-0.14**	**-0.17**
Slope 3	**REF**	-0.14	**-0.50**	-0.17	-0.19	-0.22	-0.27	-0.37
Slope 4	**REF**	**0.02**	**0.02**	**0.01**	0.01	0.01	0.01	0.02
Maximum	**REF**	**-15.8**	**-17.9**	**-18.2**	**-22.9**	**-25.9**	**-29.3**	**-32.6**

Table 5. ANOVA test coefficients when comparing NHANES education level responses to our metrics. Coefficients with corresponding p-values less than 0.05 are in bold.

Metric	Less than 9th Grade	9th Grade to 11th Grade	High School Graduate	Some College or AA Degree	College Graduate or Above
BP 1	**REF**	0.01	**0.03**	0.02	-0.02
BP 2	**REF**	**0.79**	**0.84**	**0.84**	**0.94**
BP 3	**REF**	0.03	0.06	-0.17	0.08
Slope 1	**REF**	-0.05	0.08	0.06	0.16
Slope 2	**REF**	-0.01	**-0.02**	**-0.03**	**-0.06**
Slope 3	**REF**	0.00	0.01	0.01	-0.22
Slope 4	**REF**	0.01	0.01	**0.02**	**0.02**
Maximum	**REF**	-0.48	-0.05	1.72	**6.24**

Table 6. ANOVA test coefficients when comparing NHANES household size responses to our metrics. Coefficients with corresponding p-values less than 0.05 are in bold.

Metric	1	2	3	4	5	6	7+
BP 1	**REF**	**-0.04**	**-0.06**	**-0.11**	**-0.11**	**-0.11**	**-0.10**
BP 2	**REF**	0.14	**0.62**	**0.89**	**0.82**	**0.75**	**0.64**
BP 3	**REF**	1.06	**3.26**	**4.49**	**4.61**	**4.48**	**4.64**
Slope 1	**REF**	**-0.27**	**-0.65**	**-0.98**	**-0.98**	**-1.06**	**-1.10**
Slope 2	**REF**	0.00	**0.03**	**0.04**	**0.05**	**0.06**	**0.06**
Slope 3	**REF**	-0.18	0.09	0.12	0.00	0.13	0.12
Slope 4	**REF**	0.00	-0.01	-0.01	-0.01	**-0.01**	-0.01
Maximum	**REF**	**2.93**	**9.34**	**14.0**	**16.0**	**16.6**	**15.4**

1.4 Appendix D: Weekday vs Weekend Behaviours

Given the differences in routines many individuals have between weekdays and weekends, one would expect the day of the week to influence energy expenditure. It has been shown that adults using the built environment gain different benefits depending on the day of the week[14]. Employees who walk to work have greater physical activity levels than those who drive, but this is not apparent when comparing only weekend physical activity[15]. When examining studies based on children, there are some that conclude children have higher physical activity on weekends than weekdays[16] as well as others which conclude the opposite[17].

To compare how well our metrics differentiate between weekday physical activity and weekend physical activity, both the NHANES and the INTERACT datasets were divided into weekdays and weekends. From each of these four new datasets, we recalculated a set of corresponding metrics using the same process that was used for the original two datasets. Figure 5 shows the distributions of all participants in the INTERACT dataset for their weekday and weekend data, while Figure 6 shows the same for the NHANES dataset. Table 7 shows how many participants have each profile for the weekday and weekend datasets of both INTERACT and NHANES. In both datasets, the number of participants with a non-vigorous profile is much higher on the weekends, with a larger portion of the consistent and moderately active weekday participants being less active on weekends when compared to the number of extremely active participants that are less active on weekends.

Table 7. Participant activity intensity profiles as data is separated into weekday and weekend data

	Non-Vigorous	Consistent	Moderately Active	Extremely Active	Outlier
INTERACT Weekend	371	18	44	17	63
INTERACT Weekday	299	50	99	20	45
NHANES Weekend	5639	271	1658	45	18
NHANES Weekday	4591	309	2754	66	6

1.5 Appendix E: Computation Runtime

To demonstrate the expected runtime of the model and various steps in its computation, the script was run on a desktop PC with 15 GB RAM and an Intel Core i7-8700 processor, using the NHANES dataset. For each participant, the following steps were performed and timed in order: loading the participant's data file, preprocessing the data, filtering out participants with insufficient data, sorting each minute's data points into data bins, computing the participant's metrics assuming a 4-segment line is used, and computing the participant's metrics assuming a 3-segment line is used. Participants who are filtered out are still included in the data filtering step and all previous steps mentioned above. The median participant runtime values are reported in Table 8. The largest bottleneck in the process was loading the participant's data into memory, followed by data preprocessing, then fitting the 4-segment line model and computing the resulting metrics, then binning the data points, then filtering insufficient data, and finally fitting the 3-segment line model and computing the resulting metrics.

All distributions in this section are displayed with a logarithmic y-axis. Figure 7a shows the distribution of all runtimes of all participants for the NHANES dataset, including those that are filtered out, while Figure 7b displays only those participants who are not filtered out at any stage. We clearly see this difference as there are a small number of runtimes well below the median runtime in Fig. 7a that are not present in Fig. 7b.

Table 8. NHANES mean runtime per participant

	Data Load Time	Data Prepro-cessing	Data Filtering	Data Binning	3 segment metrics	4 segment metrics	Total Median Runtime
Time (ms)	29.2	23.8	19.4	22.2	14.4	21.8	130.8

References

[1] Castañeda-Babarro, Arkaitz; Arbillaga-Etxarri, Ane; Gutiérrez-Santamaría, Borja, and Coca, Aitor. "Physical Activity Change during COVID-19 Confinement". In: *International Journal of Environmental Research and Public Health* 17.18 (2020). ISSN: 1660-4601. DOI: 10.3390/ijerph17186878. URL: https://www.mdpi.com/1660-4601/17/18/6878.

[2] Maugeri, Grazia et al. "The impact of physical activity on psychological health during Covid-19 pandemic in Italy". In: *Heliyon* 6.6 (2020), e04315. ISSN: 2405-8440. DOI: https://doi.org/10.1016/j.heliyon.2020.e04315. URL: https://www.sciencedirect.com/science/article/pii/S2405844020311592.

[3] Robinson, Eric et al. "Obesity, eating behavior and physical activity during COVID-19 lockdown: A study of UK adults". In: *Appetite* 156 (2021), p. 104853. ISSN: 0195-6663. DOI: https://doi.org/10.1016/j.appet.2020.104853. URL: https://www.sciencedirect.com/science/article/pii/S0195666320310060.

[4] Stockwell, Stephanie et al. "Changes in physical activity and sedentary behaviours from before to during the COVID-19 pandemic lockdown: a systematic review". In: *BMJ Open Sport & Exercise Medicine* 7.1 (2021). DOI: 10.1136/bmjsem-2020-000960. eprint: https://bmjopensem.bmj.com/content/7/1/e000960.full.pdf. URL: https://bmjopensem.bmj.com/content/7/1/e000960.

[5] Tison, Geoffrey H et al. "Worldwide effect of COVID-19 on physical activity: a descriptive study". In: *Annals of internal medicine* 173.9 (2020), pp. 767–770.

[6] Xu, Selene Yue et al. "Statistical approaches to account for missing values in accelerometer data: Applications to modeling physical activity". In: *Statistical Methods in Medical Research* 27.4 (2018). PMID: 27405327, pp. 1168–1186. DOI: 10.1177/0962280216657119. eprint: https://doi.org/10.1177/0962280216657119. URL: https://doi.org/10.1177/0962280216657119.

[7] Lee, Jung Ae and Gill, Jeff. "Missing value imputation for physical activity data measured by accelerometer". In: *Statistical Methods in Medical Research* 27.2 (2018), pp. 490–506. DOI: 10.1177/0962280216633248. eprint: https://doi.org/10.1177/0962280216633248. URL: https://doi.org/10.1177/0962280216633248.

[8] Kang, Minsoo; Bjornson, Kristie; Barreira, Tiago V; Ragan, Brian G, and Song, Kit. "The minimum number of days required to establish reliable physical activity estimates in children aged 2–15 years". In: *Physiological Measurement* 35.11 (Oct. 2014), pp. 2229–2237. DOI: 10.1088/0967-3334/35/11/2229. URL: https://doi.org/10.1088/0967-3334/35/11/2229.

[9] Hislop, Jane et al. "An investigation into the minimum accelerometry wear time for reliable estimates of habitual physical activity and definition of a standard measurement day in pre-school children." In: *Physiological Measurement* 35.11 (Oct. 2014), pp. 2213–2228. DOI: 10.1088/0967-3334/35/11/2213. URL: https://doi.org/10.1088/0967-3334/35/11/2213.

[10] Brown, Wendy J.; Heesch, Kristiann C., and Miller, Yvette D. "Life Events and Changing Physical Activity Patterns in Women at Different Life Stages". In: *Annals of Behavioral Medicine* 37.3 (June 2009), pp. 294–305. ISSN: 0883-6612. DOI: 10.1007/s12160-009-9099-2. eprint: https://academic.oup.com/abm/article-pdf/37/3/294/22108874/12160_2009_article_9099.pdf. URL: https://doi.org/10.1007/s12160-009-9099-2.

[11] Schmitz, Katie; French, Simone A., and Jeffery, Robert W. "Correlates of Changes in Leisure Time Physical Activity over 2 Years: The Healthy Worker Project". In: *Preventive Medicine* 26.4 (1997), pp. 570–579. ISSN: 0091-7435. DOI: https://doi.org/10.1006/pmed.1997.0178. URL: https://www.sciencedirect.com/science/article/pii/S009174359790178X.

[12] Weiss, Deborah R; O'Loughlin, Jennifer L; Platt, Robert W, and Paradis, Gilles. "Five-year predictors of physical activity decline among adults in low-income communities: a prospective study". In: *International Journal of Behavioral Nutrition and Physical Activity volume* 4.2 (2007). DOI: https://doi.org/10.1186/1479-5868-4-2.

[13] Van Dyck, Delfien et al. "Moderating effects of age, gender and education on the associations of perceived neighborhood environment attributes with accelerometer-based physical activity: The IPEN adult study". In: *Health & Place* 36 (2015), pp. 65–73. ISSN: 1353-8292. DOI: https://doi.org/10.1016/j.healthplace.2015.09.007. URL: https://www.sciencedirect.com/science/article/pii/S1353829215001288.

[14] Cerin, Ester et al. "Do associations between objectively-assessed physical activity and neighbourhood environment attributes vary by time of the day and day of the week? IPEN adult study". In: *International Journal of Behavioral Nutrition and Physical Activity* 14.1 (Mar. 2017), p. 34. ISSN: 1479-5868. DOI: 10.1186/s12966-017-0493-z. URL: https://doi.org/10.1186/s12966-017-0493-z.

[15] Audrey, Suzanne; Procter, Sunita, and Cooper, Ashley R. "The contribution of walking to work to adult physical activity levels: a cross sectional study". In: *International Journal of Behavioral Nutrition and Physical Activity* 11.1 (Mar. 2014), p. 37. ISSN: 1479-5868. DOI: 10.1186/1479-5868-11-37. URL: https://doi.org/10.1186/1479-5868-11-37.

[16] Peiró-Velert, Carmen; Devís-Devís, Jose; Beltrán-Carrillo, Vicente J., and Fox, Kenneth R. "Variability of Spanish adolescents' physical activity patterns by seasonality, day of the week and demographic factors". In: *European Journal of Sport Science* 8.3 (2008), pp. 163–171. DOI: 10.1080/17461390802020868. eprint: https://doi.org/10.1080/17461390802020868. URL: https://doi.org/10.1080/17461390802020868.

[17] Fairclough, Stuart J.; Boddy, Lynne M.; Mackintosh, Kelly A.; Valencia-Peris, Alexandra, and Ramirez-Rico, Elena. "Weekday and weekend sedentary time and physical activity in differentially active children". In: *Journal of Science and Medicine in Sport* 18.4 (2015), pp. 444–449. ISSN: 1440-2440. DOI: https://doi.org/10.1016/j.jsams.2014.06.005. URL: https://www.sciencedirect.com/science/article/pii/S1440244014001169.

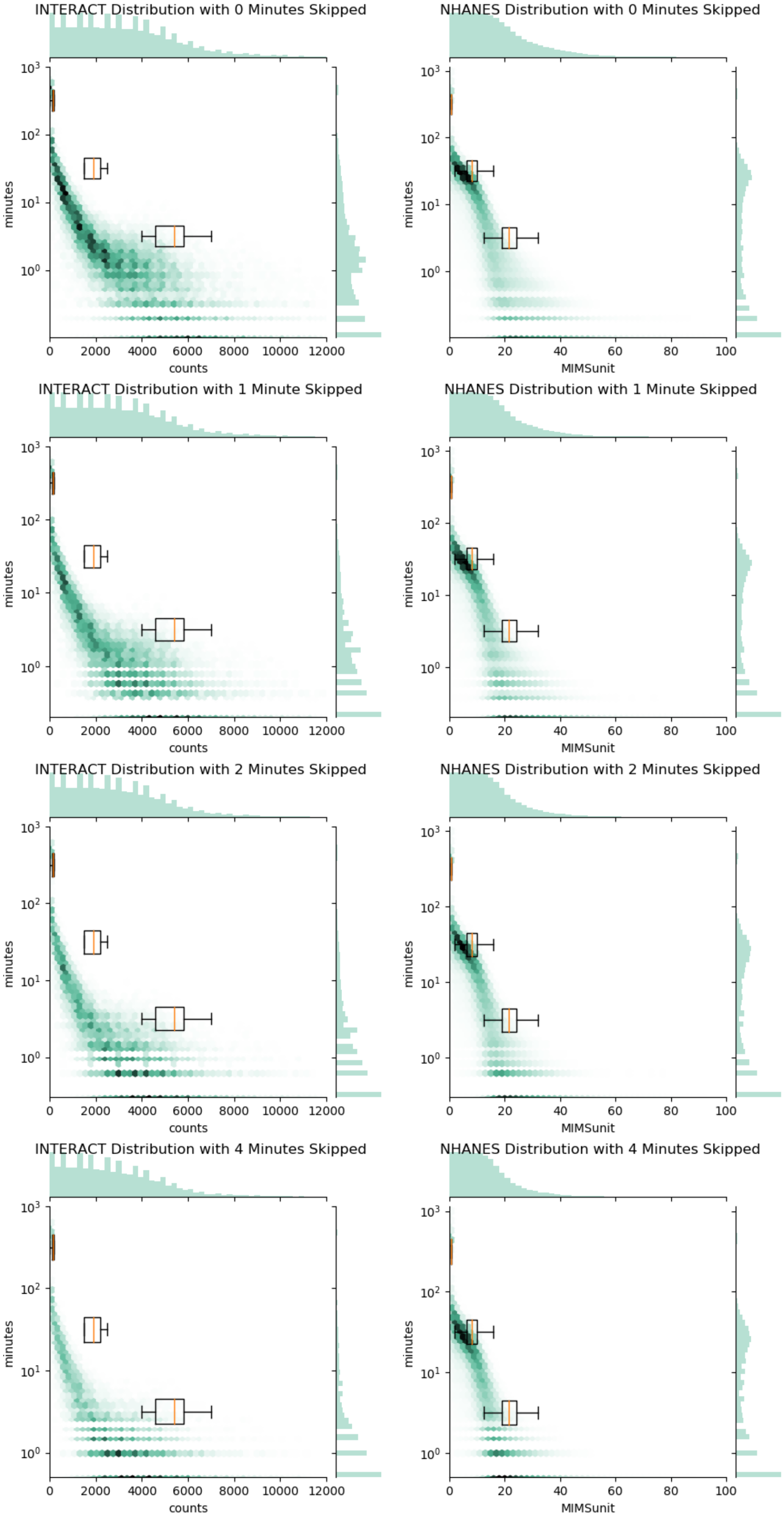

Figure 2. The distributions of all participants for the NHANES and INTERACT studies with varying minutes skipped per cycle. In order, the number of minutes skipped for each row is 0, 1, 2, and 4.

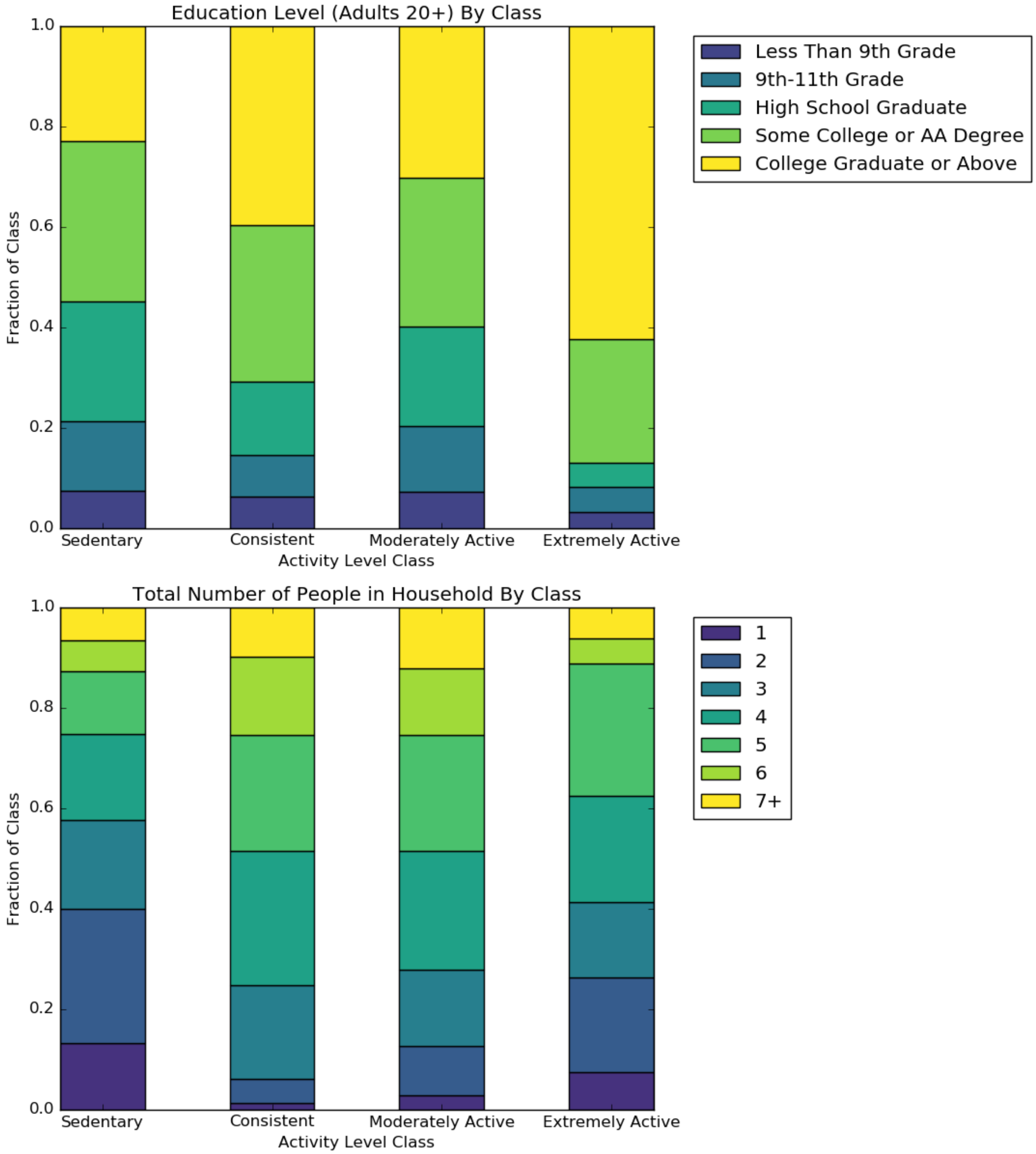

Figure 3. The breakdown of education levels (a) and household sizes (b) for each activity intensity profile with the NHANES dataset. As each profile is of a different size, the bars represent what portion of that profile gave that response, rather than the total number of individuals.

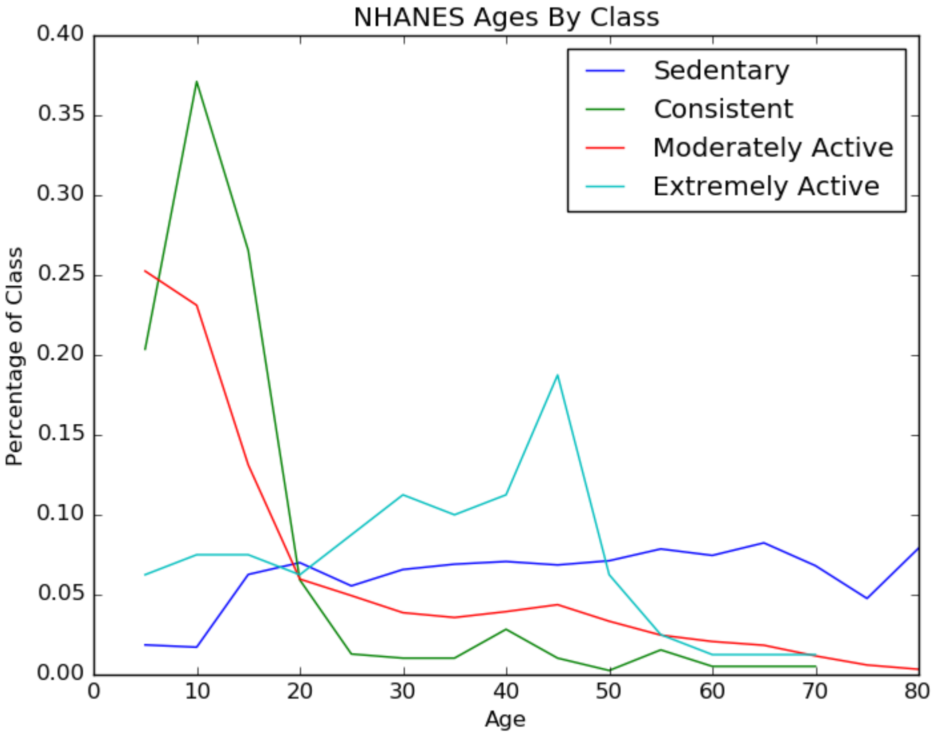

Figure 4. The breakdown of ages for each activity intensity profile with the NHANES dataset. As each profile is of a different size, the lines represent what portion of that profile gave that response, rather than the total number of individuals.

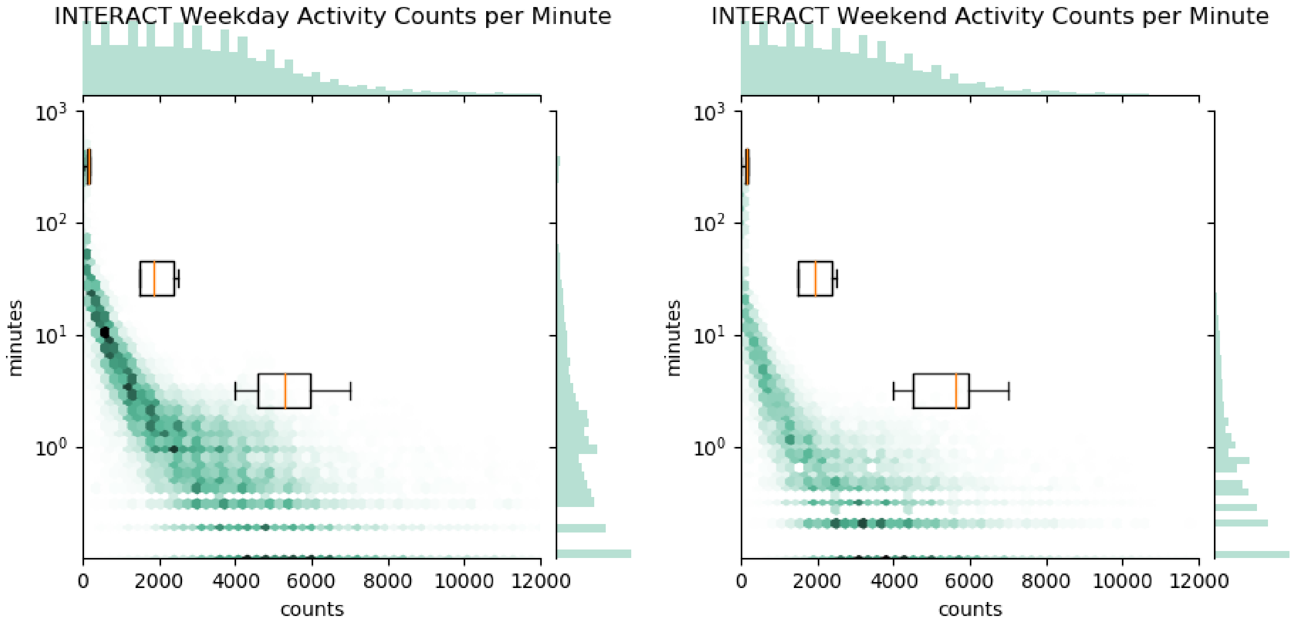

Figure 5. A comparison of the distributions of activity counts between INTERACT's weekday entries and weekend entries.

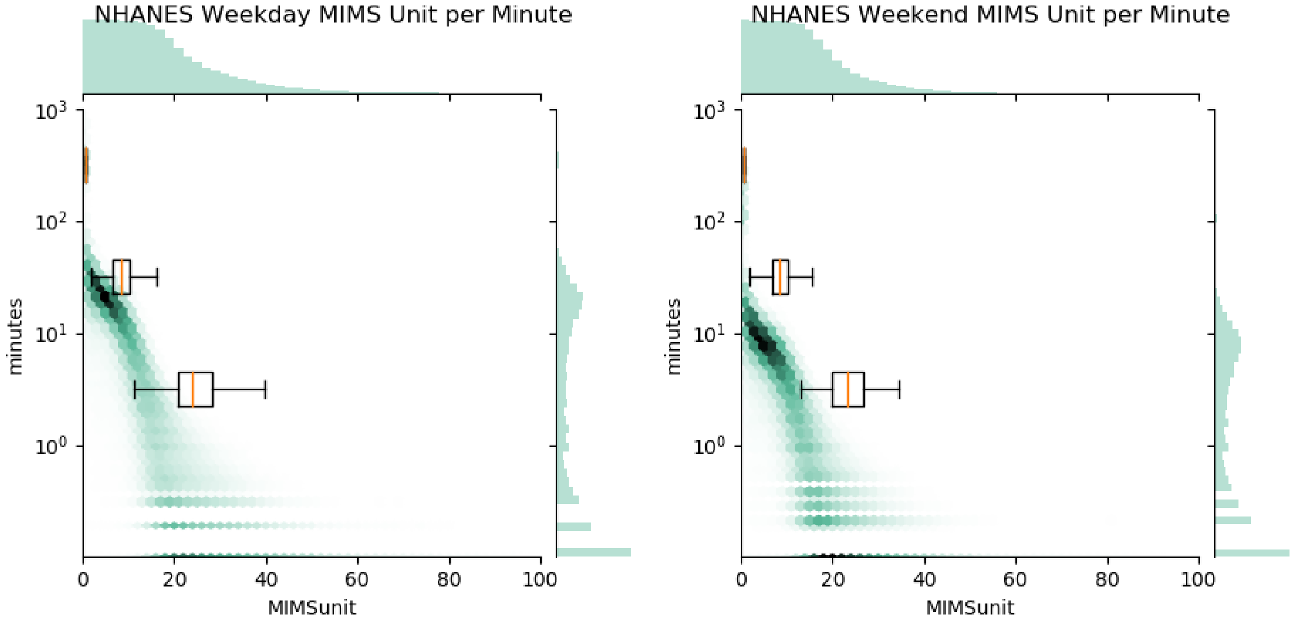

Figure 6. A comparison of the distributions of activity counts between NAHNES's weekday entries and weekend entries.

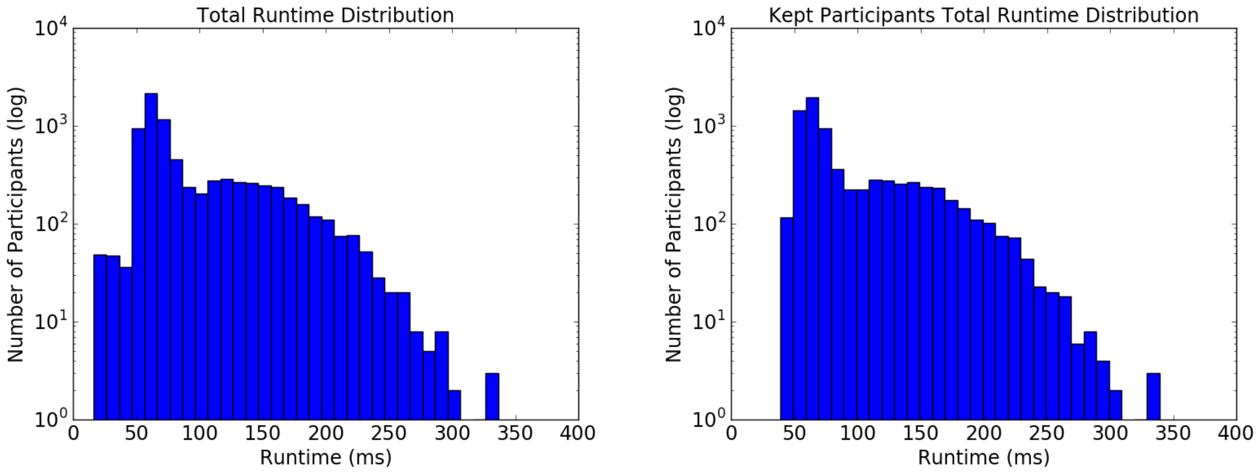

Figure 7. Computational runtime distributions for the NHANES participants. The first histogram (a) shows the runtime distributions for all participants, while the second (b) excludes participants who are filtered out at any stage.
